# Preclinical Safety Evaluation of *Leuconostoc lactis* DMLL10 Isolated from Kimchi in Rats

**DOI:** 10.4014/jmb.2601.01021

**Published:** 2026-04-10

**Authors:** Hee-Jung Park, Sei Park, Sojeong Heo, Do-Won Jeong

**Affiliations:** 1 Department of Foodservice Management and Nutrition, Sangmyung University, Seoul 03016, Republic of Korea; 2 Department of Food and Nutrition, Dongduk Women’s University, Seoul 02748, Republic of Korea

**Keywords:** *Leuconostoc lactis* DMLL10, Oral toxicity, Acute, Repeated-dose study

## Abstract

*Leuconostoc lactis* DMLL10 is a novel strain isolated from kimchi. The strain did not exhibit toxicity in genetic tests. This study aims to conduct a preclinical safety assessment by performing repeated-dose toxicity studies in animals. In the acute toxicity test, rats were administered 0 or a daily dose of 6,250 mg/kg/day of *Leu. lactis* DMLL10. In the 90-day repeated-dose study, daily doses of 0, 1,250, 2,500, and 5,000 mg/kg/day were given to rats. Body weight (BW), relative organ weights (ROW), hematological, biochemical, and pathological parameters were analyzed, and daily clinical signs were observed. The acute toxicity trial showed that the maximum tolerated dose of *Leu. lactis* DMLL10 was greater than 6,250 mg/kg/day. In the acute and subchronic trials, no significant changes were observed in BW, ROW, hematological parameters, or biochemical biomarkers when *Leu. lactis* DMLL10 was administered orally at a dose of 5,000 mg/kg/day. These results indicate that oral administration of *Leu. lactis* DMLL10 at doses up to 5,000 mg/kg/day is considered safe in rats, providing a basis for the clinical use of *Leu. lactis* DMLL10.

## Introduction

Kimchi is a representative Korean fermented food made primarily from vegetables such as napa cabbage and radish, possessing nutritional characteristics that are low in calories and rich in dietary fiber, vitamin C, β-carotene, vitamin A precursors, vitamin K, and folate [1]. The insoluble and soluble dietary fiber derived from vegetables promotes intestinal motility and increases satiety [2-4]. Some studies report that kimchi consumption may be associated with improved blood lipid levels, reduced insulin resistance, and decreased body fat, among other metabolic health indicators, making it noteworthy for preventing metabolic syndrome. It also contains minerals like potassium, calcium, and iron, along with diverse phytochemicals such as capsaicin from chili peppers, sulfur compounds from garlic and scallions, and glucosinolate breakdown products derived from cabbage. These components offer potential physiological activity related to antioxidant and anti-inflammatory effects.

During kimchi fermentation, lactic acid bacteria such as *Lactiplantibacillus plantarum*, *Leuconostoc* (*Leu.*) *mesenteroides* and *Leu. lactis* proliferates, potentially improving gut microbial balance and inhibiting harmful bacteria. Lactic acid bacteria and fermentation metabolites derived from kimchi are reported to contribute to immune function regulation by participating in maintaining intestinal mucosal function and modulating immune cell activity [5-7]. Furthermore, the fermentation process is suggested to increase the bioavailability of compounds like polyphenols and enhance antioxidant activity, potentially exerting positive effects on alleviating oxidative stress and chronic inflammatory responses [8, 9].

*Leu. lactis* is already listed in the Qualified Presumption of Safety (QPS) and the International Dairy Federation (IDF) and is recognized as a non-pathogenic microorganism [10-14]. No toxicity was observed in our genotoxicity study [15]. In a previous study, we demonstrated the genetic stability and lack of mutagenicity of *Leu. lactis* DMLL10, indicating its non-toxicity and potential as a dependable and safe functional food ingredient. After confirming the genotoxic safety of probiotics, strain-level toxicological verification in rodents is essential for regulatory approval and commercialization. Acute toxicity testing in animals generally refers to examining whether harmful effects occur following a single exposure to a substance over a short period, typically within 24 h. The severity of adverse effects caused by a new substance is assessed based on the concentration that induces specific health abnormalities leading to death, typically using indicators such as LD50 (lethal dose causing death in 50% of the test population) or LC50 (lethal concentration causing death in 50% of the test population) [16]. For chronic toxicity, it is a subchronic toxicity test where the substance is administered orally to test animals (primarily rodents) daily for 90 days [17]. Therefore, this study aims to perform acute and subchronic toxicity assessments of *Leu. lactis* DMLL10. This can contribute to the safety evaluation of chemicals, pharmaceuticals, and food additives by providing detailed information on the toxicological profile of the test substance.

## Materials and Methods

### Sample Preparation

*Leu. lactis* DMLL10 was isolated from kimchi [18]. The strain was cultured in 500 L of food-grade medium (2% glucose, 1% yeast extract, 1% soy peptone, 0.5% CH_3_COONa, 0.2% K_2_HPO_4_, 0.01% MgSO_4_, 0.005% MnSO_4_) at 30°C for 18 h. After incubation, the medium was centrifuged at 7,500 × g for 10 min, the supernatants were discarded, and the cell pellets were freeze-dried and stored at −20°C until use. The final concentration was 6.8 × 10^10^ CFU/g.

The test material was weighed and placed into a preparation container. An excipient (DPBS, Dulbecco's Phosphate-Buffered Saline, USA) was added, and the mixture was suspended using a stirrer. Stirring was performed at 700 rpm or below. The formulation was prepared on the day of administration, immediately prior to administration.

### Experimental Animals Housing

Sprague–Dawley (SD) rats (ORIENTBIO INC., Republic of Korea), 6 weeks of age, were used for the single-dose, 14-day repeated-dose, and 90-day repeated-dose oral toxicity studies. A total of 10 males and 10 females (137.2–157.6 g for males, 118.6–129.1 g for females) were used for the single-dose study; 20 males and 20 females (102.7–149.0 g) for the 14-day repeated-dose study; and 40 males and 40 females (159.7–238.8 g) for the 90-day repeated-dose study. The animals were housed individually in plastic cages by sex under controlled environmental conditions (temperature: 19.0–25.0°C; relative humidity: 38.5–68.1%; 12 h light/dark cycle, lights on from 7:00 a.m. to 7:00 p.m.; illumination: 150–300 lux). All animals were provided with a Teklad Certified Irradiated Global 18% Protein Rodent Diet 2918C (Envigo RMS, Inc., USA) and drinking water ad libitum throughout the study period.

### Experimental Animal Designs

All experiments were conducted in compliance with the Animal Protection Act (Law No. 4379) and were approved by the Biotoxtech Animal Ethics Committee. The toxicity of *Leu. lactis* DMLL10 in acute (Approval no. 220661), 14-days repeated dose (Approval no. 220729), and subchronic toxicity tests (Approval no. 230056) were performed in accordance with GLP regulations at Biotoxtech (Republic of Korea). The acute oral toxicity of *Leu. lactis* DMLL10 was evaluated in rat according to the method specified in OECD Guideline No. 423 [19]. A 14-day repeated-dose preliminary study was conducted for dose selection, with reference to the general principles of OECD TG 407 [20]. The 90-day repeated-dose oral toxicity study was performed according to OECD Guideline No. 408 [17]. The relevant experimental conditions are presented in Table 1.

In an acute toxicity test, the rat was given orally once daily at a dose of 6,250 mg/kg (1.0 × 10^11^ CFU/kg) or DPBS. On the day of administration (Day 1), observations were made approximately 30 min after administration, and again at approximately 1, 2, 4, and 6 h. General condition (type of toxic signs, onset time, recovery time, etc.) and mortality were observed. Body weight was measured on the day of administration (pre-administration), and on days 2, 4, 8, and 15 (necropsy day). All animals were euthanized on the necropsy day by CO2 inhalation followed by bleeding from the abdominal aorta and then necropsied. No gross pathological findings were observed during necropsy, so histopathological examination was not performed.

For the 90-day repeated toxicity study, the SD rats were randomly divided into a control group (G1) and three treatment groups (G2~G4, n = 20; 10 males and 10 females per group). *Leu. lactis* DMLL10 was administered once daily via oral gavage for 90 sequential days at doses of 1,250 (2 × 10^10^ CFU/kg/day), 2,500 (4 × 10^10^ CFU/kg/day), and 5,000 mg/kg/day (8 × 10^10^ CFU/kg/day). The control group received only DPBS. During the observation period, general symptoms were observed once daily for all animals, and the presence of moribund or dead animals was checked twice daily. All animals were euthanized by bleeding from the abdominal aorta under isoflurane anesthesia on the day of necropsy and subsequently necropsied. A detailed macroscopic examination of all systemic organs and tissues was performed on all animals necropsied.

### Dose Selection

In a preliminary 14-day repeated oral dose toxicity study of *Leu. lactis* DMLL10 at doses of 1,250, 2,500 and 5,000 mg/kg/day. Forty SD rats were randomly assigned to one control group (G1) and three treatment groups (G2~G4, n = 10; 5 males and 5 females per group). Throughout the 14-day study, all experimental animals were observed twice daily for general symptoms, changes in body weight, mortality, and any signs of gross toxicity. At the end of the drug administration period (over 18 h after the final dose), all surviving rats in each group were euthanized for analysis of hematological and biochemical indicators, histopathology, and relative organ weight (ROW) [21, 22]. The dose selection for the 90-day repeated-dose toxicity study was based on the results of a preliminary repeated-dose study conducted to identify the dose range. No treatment related adverse effects were observed in terms of body weight (Table S1), organ weights (Table S2), hematology test (Table S3), and clinical chemistry (Table S4) at any dose tested. Based on these results, the maximum dose at which no signs of systemic toxicity were observed was determined to be 5,000 mg/kg/day. Using a factor of 2, the low dose and medium dose were set at 1,250 mg/kg/day and 2,500 mg/kg/day, respectively. The control group received the same volume of vehicle as the test substance groups.

### Body Weight and Food Consumption for 90 Days

Body weight was measured on the start date of administration (Day 1 of administration, pre-administration), once weekly after the start of administration, and on the necropsy date. However, the necropsy date weight was excluded from the weight assessment due to fasting. Pre-administration feed intake was measured as the daily intake from the group separation date to the start date of administration. During the treatment period, feed intake was measured weekly, and the average daily intake was calculated. Individual feed intake was calculated by dividing the total feed intake per cage by the number of animals per cage.

### Organ Weight Measurement

For all animals, the wet weight of the following organs was measured, and the relative organ weight ratio to fasting body weight was calculated. For paired organs, the weights of both sides were combined. Measured organs included the brain, pituitary gland, thyroid gland with parathyroid gland, heart, thymus, liver, spleen, adrenal gland, kidney, testis, epididymis, prostate gland and seminal vesicle with coagulating gland, uterus with cervix, and ovary.

### Hematological and Biochemical Analysis

Upon completion of the trial, all the surviving rats were deprived of food for 18 h (with water permitted) before blood sampling. Blood was collected into EDTA-containing tubes for hematology. Blood profiles such as red blood cell count (RBC), white blood cell count (WBC), hematocrit (HCT), hemoglobin (HGB), mean corpuscular hemoglobin (MCH), mean corpuscular volume (MCV), mean corpuscular hemoglobin concentration (MCHC), platelet count (PLT), neutrophils (Neu), lymphocytes (Lym), monocytes (Mono), eosinophils (Eos), basophils (Baso), and reticulocytes (Reti) were measured using an animal blood cell analyzer (XN-V, SYSMEX, Japan). For coagulation testing, approximately 2 ml of collected blood was placed into a tube containing 3.2% sodium citrate. After centrifugation at 3,000 rpm for 10 min, the plasma was collected. Prothrombin time (PT) and activated partial thromboplastin time (APTT) parameters were measured using a coagulation analyzer (ACL TOP 350, Werfen, USA).

For blood biochemical testing, blood was centrifuged at 3,000 rpm for 10 min, and serum was collected. The parameters, including albumin (Alb), alanine aminotransferase (ALT), alkaline phosphatase (ALP), aspartate aminotransferase (AST), γ-glutamyl transpeptidase (GGT), total bilirubin (T-Bili), blood urea nitrogen (BUN), urea, creatinine, total bile acid (TBA), total protein (TP), albumin/globulin ratio (A/G ratio), total cholesterol (T-Chol), low-density lipoprotein cholesterol (LDL-C), high-density lipoprotein cholesterol (HDL-C), triglycerides (TG), glucose (Glu), phosphorus, calcium, chloride, sodium, and potassium were measured using a blood biochemistry analyzer (Labospect 006, Hitachi, Japan). Hormone tests were performed using serum from the blood biochemical test sample (stored at -80 to -60°C until analysis) using an immunoassay analyzer (IMMULITE 1000, Siemens, Germany) and an ELISA reader (ELx808, BioTek, USA) to measure triiodothyronine (T3) and total thyroxine (T4), and thyroid-stimulating hormone (TSH).

### Histopathology

For all animals subjected to necropsy, all organs and tissues from control and high-dose groups (5,000 mg/kg/day) were removed and fixed in 10% neutral buffered formalin solution. These organs were brain, pituitary gland, thyroid gland, parathyroid gland, thymus, lung with bronchus, heart, aorta, trachea, spleen, liver, adrenal gland, kidney, salivary gland, esophagus, stomach, duodenum, jejunum, ileum with Peyer’s patches, cecum, colon, rectum, pancreas, testis, epididymis, prostate gland, seminal vesicle, coagulating gland, ovary, uterus with cervix, vagina, urinary bladder, sciatic nerve, mesenteric lymph node, submandibular lymph node, eye, optic nerve, harderian gland, skin (inguinal), mammary gland (inguinal), bone, bone marrow, skeletal muscle (biceps femoris), spinal cord, and tongue. Among these, the testis, eyes, and optic nerves were first fixed in Davidson's fixative before being fixed in 10% neutral buffered formalin solution. The fixed tissue was embedded in paraffin and then stained with hematoxylin and eosin for microscopic examination.

### Statistical Analysis

For the 90-day repeated-dose study, the mean and standard deviation were also calculated for each group. Statistical analyses were performed using Provantis^TM^ (Instem plc., UK). Bartlett’s test was used to examine homogeneity of variance among groups. If variance was homogeneous (*p* ≥ 0.05), one-way ANOVA was conducted, followed by Dunnett’s test for pairwise comparisons when significant differences were detected (*p* < 0.05). If Bartlett’s test indicated heterogeneous variance (*p* < 0.05), the Kruskal–Wallis test was applied to rank-transformed data, and when significant group differences were observed (*p* < 0.05), Dunn’s test was performed for pairwise comparisons.

## Results

### Acute Toxicity

**Fig. 1:**
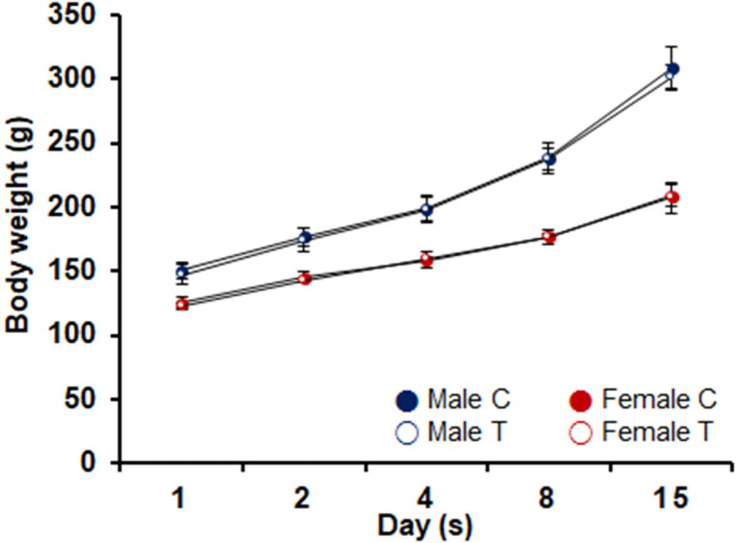
Body weight changes of experimental animal by group in acute experiment. C: control; T: *Leuconostoc lactis* DMLL10 administered at 6,500 mg/kg/day.

No deaths were observed in any animals, male or female, during the observation period. No abnormalities in general symptoms were observed in any animals, male or female, during the observation period. During the observation period, no statistically significant changes in body weight were observed in the 6,250 mg/kg treatment group compared to the control group (Fig. 1). No abnormalities were observed in the gross findings at necropsy in any animals of either sex (data not shown).

### Clinical Observations and Mortality for 90-Day Toxicity Trial

No treatment-related deaths were observed in any dose group throughout the entire experimental period. Furthermore, no abnormal clinical signs or obvious toxic effects associated with *Leu. lactis* DMLL10 administration were detected in any animal, regardless of dose level or treatment duration. However, scab formation was observed in one animal each in the control group and the 2,500 mg/kg/day group. Hair loss was observed in one animal in the control group. These symptoms showed no dose-related correlation and were observed in only one animal per group. Therefore, they were judged to be incidental findings.

### Body Weight Monitoring and Food Consumption for 90-Day Toxicity Trial

**Fig. 2:**
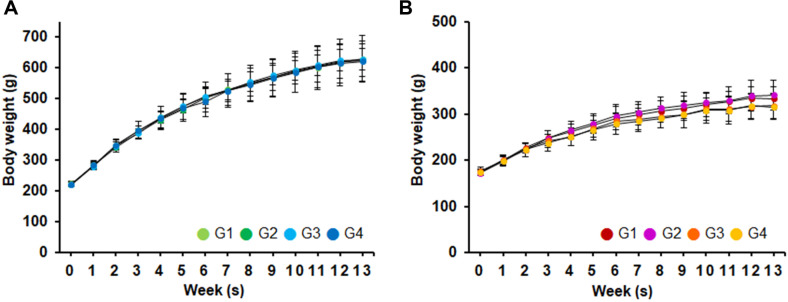
Body weight changes of experimental animal by group in the 90-day repeated oral administration study. (**A**) male, (**B**) female. G1, control; G2–G4, *Leuconostoc lactis* DMLL10 administered at 1,250, 2,500, and 5,000 mg/kg/day, respectively.

The average weight over the 90-day period is shown in Fig. 2. Body weight increased progressively in all groups throughout the study period, consistent with normal physiological growth. During the administration period, no changes in body weight attributable to the test substance were observed in the 1,250, 2,500 and 5,000 mg/kg/day treatment groups compared to the control group.

**Fig. 3:**
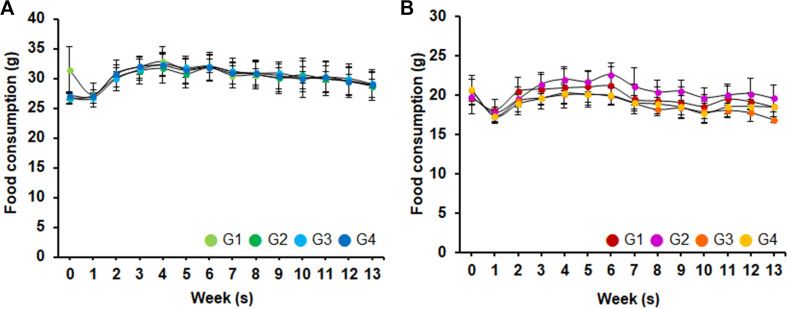
Food consumption changes observed in the 90-day repeated oral administration study. (**A**) male, (**B**) female. G1, control; G2–G4, *Leuconostoc lactis* DMLL10 administered at 1,250, 2,500, and 5,000 mg/kg/day, respectively.

Fig. 3 shows the average feed intake over 90 days. During the administration period, no significant changes in feed intake due to the test substance were observed in the 1,250, 2,500 and 5,000 mg/kg/day treatment groups compared to the control group.

### Organ Weights for 90-Day Toxicity Trial

In the 1,250, 2,500 and 5,000 mg/kg/day treatment groups, no changes in organ weights due to the test substance were observed compared to the control group (Table 2). However, a statistically significant difference in liver weight was observed in females. As this change was not dose-dependent, it was not considered to be toxicity caused by the test substance. Although statistically significant changes in thyroid and parathyroid organ weights were observed, evidence of a dose-related relationship was weak. In addition, no treatment-related alterations were detected in serum thyroid hormone levels (T3, T4, TSH). Accordingly, these findings were not considered toxicologically relevant.

### Hematology and Clinical Biochemistry for 90-Day Toxicity Trial

Hematological evaluation revealed no treatment-related changes in animals administered 1,250, 2,500 and 5,000 mg/kg/day (Table 3). Although some parameters showed statistical significance, these findings were minimal and not dose-dependent and were therefore considered incidental.

In the blood biochemistry assessments, no changes due to the test substance were observed in the male and female groups administered 1,250, 2,500 and 5,000 mg/kg/day compared to the control group (Table 4). Statistical significance observed solely in the Cl? level in female rats was regarded as incidental, due to the absence of dose dependency.

### Histopathology

Table 5 summarizes the histopathological findings observed in rats administered *Leu. lactis* DMLL10 orally for 90 days, organized by organ and sex.

In males, mild histological changes were observed in the adrenal glands, pituitary gland, thyroid gland, heart, kidneys, liver, lungs/bronchi, and small intestine (jejunum). Major findings included cortical atrophy (grade 1), pseudocysts, ultimobranchial cysts, mild inflammatory cell infiltration (grade 1), renal tubular basophilic changes, medullary cysts, mild lipodystrophy, and mononuclear cell infiltration. These findings were observed sporadically in both the control and treatment groups, and no dose-dependent increase was confirmed.

In females, mild lesions were also observed in the Harder's gland, salivary glands (submandibular glands), thyroid gland, heart, kidneys, liver, lungs/bronchi, small intestine (jejunum), and vagina. Major findings included inflammatory cell infiltration (grade 1), mononuclear cell infiltration, terminal acinar cysts, medullary cysts, mild lipodystrophy, increased macrophages, and vaginal mucification. Most changes were mild, with no significant difference in incidence or severity compared to the control group.

In organs examined histopathologically, no specific lesions related to test substance administration or noteworthy abnormal findings were observed in organs other than those listed in the table. Overall, the histopathological changes observed were judged to be spontaneous or nonspecific findings and were interpreted as unrelated to the test substance.

### Determination of NOAEL

Changes in organ weight were not considered toxicologically significant as they were not dose-dependent and showed no association with relevant histopathological findings or hormonal changes. No changes related to test substance administration were observed in hematological or serum biochemical tests. Furthermore, no clinical symptoms or deaths occurred due to test substance administration, and no toxicologically significant changes were identified in body weight or feed intake. Pathological histological examination also revealed no adverse lesions related to the test substance. Based on these combined results, the No Observed Adverse Effect Level (NOAEL) for *Leu. lactis* DMLL10 is determined to be the highest dose used in this study, 5,000 mg/kg/day.

## Discussion

This study was performed to investigate NOAEL and evaluate the systemic toxicity for the *Leu. lactis* DMLL10 from Kimchi, on male and female Sprague-Dawley rats. A 90-day repeated-dose oral toxicity study was performed using gavage administration at a dose level of 0 (vehicle control), 1,250 mg/kg/day (low dose group), 2,500 mg/kg/day (middle dose group), and 5,000 mg/kg/day (high dose group).

Body weight is regarded as a highly sensitive and integrative indicator of overall systemic toxicity, with a decrease of approximately 5% commonly accepted as a threshold associated with pathological significance [23]. In this experiment, body weight increased progressively in all groups, consistent with normal physiological growth and no significant differences in body weight changes were observed between the treated group and the control group. No significant differences in body weight were observed between the treatment group and the control group, and no evidence of a dose-dependent effect was found. These results suggest that *Leu. lactis* DMLL10 did not adversely affect growth or metabolic homeostasis during the study period.

Organ weight or organ weight ratio are also widely used as a reliable indicator of potential target organ toxicity, particularly when interpreted in conjunction with body weight and histopathological findings [24-26]. In this study, no absolute or relative changes in organ weights related to the test substance were observed in most dose groups. No significant changes were observed in organ weights or organ-to-body weight ratios, and no test substance-related effects were identified in body weight changes.

Hematological and biochemical assessments are commonly used to evaluate potential effects on hematopoietic function and major organs such as the liver and kidneys [27–30]. Changes in these parameters reflect toxic effects and functional abnormalities caused by the test substance. In the present study, all measured values were comparable between the treated and control groups and showed no dose-dependent trends. These findings indicate the absence of systemic toxicity associated with repeated oral administration of *Leu. lactis* DMLL10.

Histopathological examination revealed no test substance-related lesions in any of the major organs (liver, kidneys, heart, lungs, spleen, and reproductive organs). The findings observed showed no significant differences compared to the control group, and no dose-dependent pattern was observed. Some minor changes were at levels commonly observed spontaneously and were interpreted as unrelated to the administration of the test substance. Therefore, it is concluded that the test substance did not induce toxicity at the histological level.

In some clinical observations, scab formation and hair loss were observed in animals. However, these findings were observed in both the control and treatment groups. No clear dose-response relationship was observed, and the incidence rates were low and similar between the two groups. In addition, statistically significant changes in organ weights were observed, including increased liver weight and increased thyroid/parathyroid weights at some level. However, the observed changes were not considered to exhibit a dose-response relationship, and no corresponding changes were observed in relevant clinical pathological indicators such as T3, T4, and TSH. Furthermore, no histopathological changes related to the test concentration were observed in liver or thyroid/parathyroid tissues. Regarding changes in liver weight, serum biochemical markers such as ALT and AST remained within the normal range. No histopathological effects were found in the liver. This suggests that the observed changes in organ weight were not associated with functional or structural toxicity. Therefore, no related abnormalities were observed in clinical signs, body weight, hematological parameters, or histopathological findings.

In conclusion, the results of this study showed no *Leu. lactis* DMLL10-related deaths or clinical abnormalities during the acute and subchronic experimental periods. Hematological and serum biochemical data revealed no differences between the *Leu. lactis* DMLL10 treatment group and the control group. Histopathological examination also confirmed no toxic effects on vital organs or reproductive organs at any level. Therefore, NOAEL of *Leu. lactis* DMLL10 was established at the maximum experimental dose of 5,000 mg/kg/day, providing a basis for determining a reasonable safe dose for future functional ingredient development.

## Supplemental Materials

Supplementary data for this paper are available on-line only at http://jmb.or.kr.



## Figures and Tables

**Table 1:** Summary of preclinical toxicity studies of *Leuconostoc lactis* DMLL10.

Study Type	Species (Strain)	Sex	n/group	Dose Levels (mg/kg/day)	Duration
Acute toxicity	Rat (Sprague–Dawley)	Male/Female	5/sex/group	0, 6,250	Single dose
14-day repeated-dose	Rat (Sprague–Dawley)	Male/Female	5/sex/group	0, 1,250, 2,500, 5,000	14 days
90-day repeated-dose	Rat (Sprague–Dawley)	Male/Female	10/sex/group	0, 1,250, 2,500, 5,000	90 days

**Table 2:** Relative organ weight of *Leuconostoc lactis* DMLL10-administered group.

Parameter	Unit	Leuconostoc lactis DMLL10 (mg/kg/day)			
		0	1,250	2,500	5,000
No. of rats		20	20	20	20
Male					
Relative Adrenal	%	0.01 ± 0.002	0.01 ± 0.001	0.01 ± 0.002	0.01 ± 0.002
Relative Brain	%	0.38 ± 0.035	0.39 ± 0.036	0.37 ± 0.030	0.38 ± 0.040
Relative Heart	%	0.28 ± 0.023	0.27 ± 0.022	0.28 ± 0.020	0.28 ± 0.019
Relative Kidney	%	0.56 ± 0.028	0.54 ± 0.036	0.58 ± 0.051	0.58 ± 0.042
Relative Liver	%	2.60 ± 0.172	2.54 ± 0.245	2.58 ± 0.196	2.60 ± 0.185
Relative Spleen	%	0.16 ± 0.026	0.16 ± 0.030	0.17 ± 0.017	0.17 ± 0.025
Relative Thyroid/Parathyroid	%	0.0042 ± 0.001	0.0041 ± 0.001	0.0056 ± 0.001^*^	0.0051 ± 0.002
Relative Testis	%	0.63 ± 0.061	0.59 ± 0.048	0.62 ± 0.047	0.63 ± 0.052
Relative Thymus	%	0.06 ± 0.015	0.06 ± 0.013	0.07 ± 0.018	0.06 ± 0.010
Relative Epididymis	%	0.29 ± 0.026	0.28 ± 0.022	0.30 ± 0.028	0.29 ± 0.037
Relative Pituitary	%	0.0023 ± 0.000	0.0022 ± 0.000	0.0022 ± 0.000	0.0023 ± 0.000
Relative Prostate/SV/C	%	0.69 ± 0.073	0.66 ± 0.072	0.70 ± 0.074	0.69 ± 0.092
Female					
Relative Adrenal	%	0.02 ± 0.003	0.02 ± 0.004	0.02 ± 0.003	0.02 ± 0.004
Relative Brain	%	0.66 ± 0.069	0.64 ± 0.057	0.69 ± 0.057	0.68 ± 0.074
Relative Heart	%	0.32 ± 0.032	0.32 ± 0.010	0.32 ± 0.014	0.33 ± 0.029
Relative Kidney	%	0.63 ± 0.070	0.62 ± 0.034	0.61 ± 0.038	0.64 ± 0.069
Relative Liver	%	2.57 ± 0.182	2.63 ± 0.330	2.37 ± 0.099^*^	2.69 ± 0.256
Relative Ovary	%	0.03 ± 0.009	0.03 ± 0.007	0.03 ± 0.005	0.03 ± 0.004
Relative Spleen	%	0.17 ± 0.017	0.19 ± 0.014	0.18 ± 0.021	0.19 ± 0.020
Relative Thyroid/Parathyroid	%	0.0058 ± 0.002	0.0056 ± 0.001	0.0077 ± 0.001^**^	0.0074 ± 0.001^**^
Relative Thymus	%	0.09 ± 0.013	0.09 ± 0.029	0.09 ± 0.019	0.09 ± 0.021
Relative Uterus	%	0.25 ± 0.046	0.25 ± 0.085	0.29 ± 0.120	0.26 ± 0.066
Relative Pituitary	%	0.01 ± 0.002	0.01 ± 0.001	0.01 ± 0.001	0.01 ± 0.001

Values are presented as the mean ± standard deviation

Relative organ weight= (organ weight /body weight) × 100

^*^: Represents a significant difference at *p* < 0.05 level compared with the control

^**^: Represents a significant difference at *p* < 0.01 level compared with the control

**Table 3:** Effects of daily oral administration of *Leuconostoc lactis* DMLL10 for 90 days on the hematological parameters of rats.

Parameter	Units	Leuconostoc lactis DMLL10 (mg/kg/day)			
		0	1,250	2,500	5,000
No. of rats		20	20	20	20
Male					
RBC	10^5^/uL	8.5 ± 0.34	8.47 ± 0.24	8.44 ± 0.27	8.52 ± 0.29
HGB	g/dL	15 ± 0.4	15.3 ± 0.4	15.2 ± 0.5	15.2 ± 0.4
HCT	%	43.7 ± 1.2	44 ± 0.8	44.3 ± 1.3	44.2 ± 0.9
PLT	10^3^/uL	987 ± 82	938 ± 206	1026 ± 130	1000 ± 99
MCV	fL	51.5 ± 0.9	52 ± 0.9	52.5 ± 0.9	51.9 ± 1.8
MCH	pg	17.6 ± 0.4	18 ± 0.4	18 ± 0.4	17.8 ± 0.6
MCHC	g/dL	34.2 ± 0.3	34.7 ± 0.4^*^	34.3 ± 0.5	34.3 ± 0.3
Reti	%	3.37 ± 0.48	3.33 ± 0.44	3.5 ± 0.47	3.46 ± 0.62
WBC	10^3^/uL	8.36 ± 1.19	8.42 ± 1.63	9.01 ± 1.84	9.99 ± 1.62
Neu	%	16 ± 5.6	21 ± 8.2	18.9 ± 4.9	20.1 ± 5.9
Lym	%	73.5 ± 6.6	68.1 ± 9	70.7 ± 6.9	68.9 ± 6.6
Mono	%	8.3 ± 1.2	8.9 ± 2.3	8.6 ± 2.2	9 ± 1.6
Eos	%	1.8 ± 0.5	1.6 ± 0.7	1.4 ± 0.6	1.5 ± 0.4
Baso	%	0.4 ± 0.1	0.4 ± 0.1	0.4 ± 0.2	0.5 ± 0.1
PT	seconds	10.7 ± 0.7	10.8 ± 0.5	10.5 ± 0.5	10.5 ± 0.4
APTT	seconds	15.1 ± 1.9	15.7 ± 0.8	15.1 ± 1.4	15.1 ± 1.2
Female					
RBC	10^5^/uL	7.75 ± 0.3	7.61 ± 0.3	7.64 ± 0.34	7.61 ± 0.33
HGB	g/dL	14.5 ± 0.6	14.7 ± 0.6	14.7 ± 0.8	14.5 ± 0.6
HCT	%	42 ± 1.6	41.8 ± 1.7	42 ± 1.9	41.1 ± 1.4
PLT	10^3^/uL	923 ± 100	926 ± 139	922 ± 71	911 ± 109
MCV	fL	54.3 ± 1.9	54.9 ± 1.6	55 ± 1.7	54.1 ± 1.6
MCH	pg	18.8 ± 0.6	19.3 ± 0.6	19.3 ± 0.7	19 ± 0.5
MCHC	g/dL	34.6 ± 0.3	35.1 ± 0.4^*^	35.1 ± 0.4	35.2 ± 0.7^*^
Reti	%	2.83 ± 0.5	3.25 ± 0.96	3.18 ± 0.71	2.88 ± 0.68
WBC	10^3^/uL	4.32 ± 0.99	4.09 ± 0.91	3.62 ± 1.28	4.36 ± 1.38
Neu	%	15.5 ± 5.6	17.7 ± 8.4	13 ± 3.2	13.1 ± 3.8
Lym	%	74.8 ± 7.5	72.5 ± 8.8	77.4 ± 3.3	77.5 ± 3.9
Mono	%	7.4 ± 1.9	7.5 ± 1.4	7.6 ± 1.2	7.6 ± 1.5
Eos	%	1.9 ± 0.7	1.9 ± 0.7	1.6 ± 0.5	1.4 ± 0.5
Baso	%	0.4 ± 0.1	0.5 ± 0.2	0.5 ± 0.2	0.4 ± 0.2
PT	seconds	9.3 ± 0.3	9.5 ± 0.4	9.8 ± 0.4^*^	9.3 ± 0.5
APTT	seconds	15.3 ± 1	15.3 ± 0.9	15.1 ± 1.1	14.8 ± 2.1

Values are presented as the mean ± standard deviation

^*^: Represents a significant difference at *p* < 0.05 level compared with the control

**Table 4:** Effects of daily oral administration of *Leuconostoc lactis* DMLL10 for 90 days on the biochemical parameters of rats.

Parameter	Units	Leuconostoc lactis DMLL10 (mg/kg/day)			
		0	1,250	2,500	5,000
No. of rats		20	20	20	20
Male					
ALT	U/L	26.9 ± 4.9	30.1 ± 7.6	29.8 ± 9.7	27.2 ± 3.8
AST	U/L	83.3 ± 17.9	90.3 ± 26.5	93.3 ± 20.3	82.3 ± 19
ALP	U/L	262.3 ± 35.2	239.5 ± 53.1	265.8 ± 43.4	262.8 ± 74.1
GGT	U/L	0.04 ± 0.09	0.01 ± 0.02	0.01 ± 0.03	0.07 ± 0.13
TBA	μmol/L	9.3 ± 4.5	10.6 ± 8	11.9 ± 6.6	9.7 ± 4.6
TP	g/dL	5.9 ± 0.3	5.9 ± 0.5	5.9 ± 0.2	6.2 ± 0.2
Alb	g/dL	2.3 ± 0.1	2.2 ± 0.2	2.3 ± 0.1	2.4 ± 0.2
A/G ratio	ratio	0.65 ± 0.07	0.61 ± 0.05	0.64 ± 0.03	0.65 ± 0.06
BUN	mg/dL	12.5 ± 1.3	12.5 ± 1.6	12.6 ± 1.1	12.2 ± 1.7
Urea	mg/dL	27 ± 3	27 ± 3	27 ± 3	26 ± 4
Creatinine	mg/dL	0.48 ± 0.03	0.48 ± 0.04	0.49 ± 0.05	0.47 ± 0.03
T-Bili	mg/dL	0.05 ± 0.01	0.04 ± 0.01	0.05 ± 0.02	0.05 ± 0.02
T-Chol	mg/dL	78 ± 21	76 ± 15	82 ± 21	84 ± 20
HDL-C	mg/dL	21.6 ± 4.7	21.6 ± 3.4	22.2 ± 4.2	23.2 ± 3.8
LDL-C	mg/dL	4.4 ± 0.9	4.1 ± 0.8	5 ± 1.1	4.4 ± 1.5
TG	mg/dL	60 ± 28	53 ± 26	49 ± 18	65 ± 33
Glu	mg/dL	156 ± 32	156 ± 15	150 ± 18	153 ± 15
Phosphorus	mg/dL	5.78 ± 0.39	5.51 ± 0.25	5.6 ± 0.3	5.99 ± 0.39
Calcium	mg/dL	9.2 ± 0.2	9.2 ± 0.4	9.4 ± 0.2	9.4 ± 0.4
Potassium	mmol/L	4.77 ± 0.25	4.64 ± 0.24	4.61 ± 0.34	4.66 ± 0.23
Sodium	mmol/L	143.4 ± 1.1	143.3 ± 1.5	143.4 ± 0.9	143.1 ± 0.9
Chloride	mmol/L	105.9 ± 1.4	105.5 ± 1.6	105.3 ± 1.2	104.4 ± 2
T3	ng/mL	1.092 ± 0.172	1.049 ± 0.142	1.127 ± 0.087	1.118 ± 0.171
T4	ng/mL	57 ± 9.3	59.6 ± 10.3	62.6 ± 10.7	67.6 ± 11.4
TSH	ng/mL	1.141 ± 0.47	0.956 ± 0.264	1.266 ± 0.75	1.219 ± 0.485
Female					
ALT	U/L	34.4 ± 21.2	24.4 ± 8.2	29.6 ± 9.5	39 ± 23.2
AST	U/L	98.4 ± 41.4	83 ± 25.9	89.9 ± 18	106.3 ± 41.9
ALP	U/L	143.8 ± 67.7	145.3 ± 39.2	154.6 ± 26.2	154.9 ± 38.2
GGT	U/L	0.44 ± 0.19	0.34 ± 0.24	0.42 ± 0.22	0.52 ± 0.28
TBA	μmol/L	11.4 ± 4.8	12.9 ± 5	15.6 ± 11.6	17.8 ± 11.1
TP	g/dL	7.4 ± 0.4	7.4 ± 0.5	7.1 ± 0.4	7.4 ± 0.4
Alb	g/dL	3.5 ± 0.2	3.3 ± 0.3	3.2 ± 0.3	3.5 ± 0.2
A/G ratio	ratio	0.89 ± 0.07	0.83 ± 0.07	0.84 ± 0.07	0.9 ± 0.08
BUN	mg/dL	15.6 ± 2.1	15.6 ± 2	15.9 ± 2.6	15.3 ± 2.4
Urea	mg/dL	33 ± 4	33 ± 3	34 ± 6	33 ± 5
Creatinine	mg/dL	0.58 ± 0.07	0.59 ± 0.06	0.58 ± 0.06	0.56 ± 0.06
T-Bili	mg/dL	0.06 ± 0.03	0.08 ± 0.02	0.07 ± 0.02	0.08 ± 0.03
T-Chol	mg/dL	100 ± 23	93 ± 20	85 ± 8	96 ± 16
HDL-C	mg/dL	27.5 ± 4.5	26.7 ± 5	24.7 ± 2.6	27.2 ± 3.8
LDL-C	mg/dL	4.2 ± 1.2	3.8 ± 0.8	3.8 ± 0.9	3.8 ± 0.9
TG	mg/dL	21 ± 8	22 ± 12	18 ± 7	19 ± 7
Glu	mg/dL	144 ± 12	132 ± 13	134 ± 20	141 ± 25
Phosphorus	mg/dL	4.81 ± 0.9	4.82 ± 0.4	4.54 ± 0.82	4.75 ± 0.46
Calcium	mg/dL	9.9 ± 0.3	9.9 ± 0.4	9.6 ± 0.3	10 ± 0.3
Potassium	mmol/L	4.11 ± 0.27	4.11 ± 0.33	4.22 ± 0.35	4.07 ± 0.18
Sodium	mmol/L	141.9 ± 0.6	142.2 ± 0.7	142.1 ± 0.9	141.5 ± 0.7
Chloride	mmol/L	105.8 ± 0.9	105.9 ± 2.2	107.4 ± 1.1^*^	105.5 ± 1.5
T3	ng/mL	1.114 ± 0.145	1.104 ± 0.163	1.158 ± 0.104	1.115 ± 0.157
T4	ng/mL	35.2 ± 6.2	42.1 ± 11.3	39.6 ± 9.9	36.8 ± 5.7
TSH	ng/mL	2.036 ± 0.522	1.888 ± 0.713	1.86 ± 0.471	1.732 ± 0.519

Values are presented as the mean ± standard deviation

^*^: Represents a significant difference at *p* < 0.05 level compared with the control

**Table 5:** Histopathological findings of *Leuconostoc lactis* DMLL10 for 90 days.

Organ		Histopathological finding	Dose (mg/kg/day)	
			0	5,000
No. of rats			20	20
Male				
Gland	Adrenal	Cortex vacuolation, grade 1	1	1
	Pituitary	Pseudocyst, pars intermedia	0	1
	Thyroid	Ultimobranchial cyst	3	1
		Ectopic tissue(thymus)	1	1
Heart		Inflammatory cell infiltration, grade 1	1	3
Kidney		Tubular basophilia, grade 1	1	0
		Medulla cyst, hyaline grade 1	2	2
Liver		Fatty change, periportal, grade 2	2	0
		Mononuclear cell infiltration, grade 1	3	2
Lung/Bronchus		Increased macrophage grade 1	1	0
Intestine	Jejunum	Diverticulum	1	1
Female				
Gland	Harderian	Inflammatory cell infiltration, grade 1	1	2
	Salivary Parotid	Mononuclear cell infiltration, grade 1	0	1
	Thyroid	Ultimobranchial cyst	5	3
		Ectopic tissue(thymus)	1	1
Heart		Inflammatory cell infiltration, grade 1	0	1
Kidney		Medulla cyst, hyaline grade 1	0	1
		Other medulla mineralization, grade 1	0	1
Liver		Periportal fatty change, grade 2	1	0
		Mononuclear cell infiltration, grade 1	0	1
Lung/Bronchus		Increased macrophage grade 1	0	2
		Mononuclear cell infiltration, grade 1	1	0
Intestine	Jejunum	Diverticulum	1	1
Vagina		Mucification, increased grade 1	1	2

Among the organs subjects to histopathological examination, all organs other than those described had no remarkable lesion.
