# Supplementary material for: Preclinical Safety Evaluation of Leuconostoc lactis DMLL10 Isolated from Kimchi in Rats
Source: J Microbiol Biotechnol. 2026 Apr 10;36:e2601021. doi: 10.4014/jmb.2601.01021 (PMC13070967; doi:10.4014/jmb.2601.01021)
Supplement: Supplementary file 1 [file jmb-36-e2601021-supple.pdf]

J. Microbiol. Biotechnol. 2026. 36: e2601021

https://doi.org/10.4014/jmb.2601.01021

Supplementary Tables

Table S1. Changes in body weight of rats treated with a *Leuconostoc lactis* DMLL10 during a14-day period.

Parameter	*Leuconostoc lactis* DMLL10			
	0	2 × 10^{10}	4 × 10^{10}	8 × 10^{10}
No. or rats	10	10	10	10
Male				
Body weight changes (g)				
1 day	222 35 ± 8.84	222.22 ± 9.33	220.07 ± 7	222.15 ± 8.22
4 days	256.82 ± 8.25	257.31 ± 10.41	253 ± 9.57	259.34 ± 10.82
8 days	301.46 ± 7.15	301.38 ± 14.07	297.61 ± 10.06	307.39 ± 12.68
11 days	330.06 ± 10.6	333.17 ± 19.89	326.79 ± 10.54	340.61 ± 13.92
14 days	359.98 ± 11.44	360.89 ± 23.76	355.31 ± 13.33	371.24 ± 16.32
Female				
Body weight changes (g)				
1 day	178.34 ± 2.35	174.6 ± 3.75	175.88 ± 6.51	177.44 ± 2.91
4 days	191.95 ± 5.65	190.65 ± 8.6	192.54 ± 6.44	195.44 ± 4.82
8 days	212.72 ± 8.36	209.19 ± 14.64	210.34 ± 6.73	214.35 ± 6.12
11 days	224.67 ± 9.87	219.33 ± 15.18	214.32 ± 9.62	226.07 ± 4.78
14 days	239.02 ± 11.96	230.96 ± 21.26	225.5 ± 7.89	236.98 ± 8.58

Table S2. Organ weights and relative organ weights of administration group treated with *Leuconostoc lactis* DMLL10 during a14-day.

Parameter (units)		*Leuconostoc lactis* DMLL10											
		0			2×10^{10}			4×10^{10}			8×10^{10}		
No. or rats		10			10			10			10		
Male													
Terminal Body Wt.	g	324.05	±	9.27	327.28	±	20.33	323.86	±	9.42	339.01	±	15.68
Brain	g	2.0137	±	0.105	1.9767	±	0.0607	2.0676	±	0.099	2.1538	±	0.0604*
Relative Brain	%	0.6215	±	0.0286	0.6051	±	0.0244	0.6388	±	0.0332	0.6366	±	0.0392
Heart	g	1.1928	±	0.0303	1.1381	±	0.0461	1.1466	±	0.0845	1.2154	±	0.0834
Relative Heart	%	0.3682	±	0.0104	0.3483	±	0.0163	0.3541	±	0.024	0.3585	±	0.0173
Kidney	g	2.5814	±	0.1919	2.6065	±	0.1211	2.5428	±	0.2093	2.8371	±	0.1445
Relative Kidney	%	0.7971		0.0619	0.7971		0.0214	0.7844		0.047	0.8373		0.0343
Liver	g	11.146		0.5632	11.265		1.1051	10.897		0.8573	11.689		0.8224
Relative Liver	%	3.438	±	0.0788	3.4385	±	0.1696	3.3005	±	0.2011	3.4453	±	0.1713
Spleen	g	0.8246	±	0.1013	0.8226	±	0.1728	0.7094	±	0.0553	0.8808	±	0.0942
Relative Spleen	%	0.2548	±	0.0342	0.2509	±	0.0471	0.2189	±	0.0135	0.2601	±	0.0292
Female													
Terminal Body Wt.	g	221.29	±	9.87	213.83	±	17.11	207.8	±	5.12	218.48	±	6.18
Brain	g	1.896	±	0.0584	1.8569	±	0.0995	1.8943	±	0.0549	1.8667	±	0.1032
Relative Brain	%	0.8582	±	0.0472	0.8727	±	0.0817	0.9122	±	0.0376	0.8543	±	0.0395
Heart	g	0.863	±	0.0556	0.8375	±	0.0748	0.7999	±	0.0605	0.8436	±	0.0336
Relative Heart	%	0.39	±	0.0184	0.3917	±	0.0163	0.3846	±	0.0215	0.3864	±	0.0214
Kidney	g	1.8299	±	0.2114	1.712	±	0.1455	1.7643	±	0.098	1.8235	±	0.0926
Relative Kidney	%	0.8291		0.1124	0.8015		0.0842	0.8491		0.0434	0.8353		0.0516
Liver	g	7.5934		0.8316	6.7385		0.8015	7.1299		0.67	7.4728		0.4804
Relative Liver	%	3.4287	±	0.298	3.1454	±	0.1674	3.4339	±	0.345	3.4216	±	0.2225
Spleen	g	0.4936	±	0.065	0.4844	±	0.0336	0.4853	±	0.0227	0.4523	±	0.0456
Relative Spleen	%	0.2227	±	0.022	0.2267	±	0.0049	0.2338	±	0.0156	0.2073	±	0.0237

* $p < 0.05$

Table S3. Hematological values of administration group treated with *Leuconostoc lactis* DMLL10 during a14-day.

Parameter	Units	*Leuconostoc lactis* DMLL10											
		0			2 × 10^{10}			4 × 10^{10}			8 × 10^{10}		
No. or rats		10			10			10			10		
Male													
RBC	10^5/uL	6.92	±	0.45	7.02	±	0.33	6.89	±	0.34	6.65	±	0.3
HGB	g/dL	14.6	±	0.5	14.7	±	0.6	14.6	±	0.5	14.3	±	0.5
HCT	%	42.2	±	1.7	42.5	±	1.7	41.9	±	1.2	40.9	±	1.8
PLT	10^3/uL	1115	±	102	1008	±	94	1072	±	214	1040	±	139
MCV	fL	61.1	±	1.7	60.5	±	0.5	60.9	±	1.5	61.6	±	1.9
MCH	pg	21.1	±	0.8	20.9	±	0.2	21.2	±	0.8	21.5	±	0.6
MCHC	g/dL	34.5		0.4	34.6		0.3	34.8		0.5	35		0.2
WBC	10^3/uL	9.07	±	3.04	8.29	±	1.81	7.66	±	1.61	8.59	±	1.47
Female													
RBC	10^5/uL	6.96	±	0.49	7.07	±	0.46	7.62	±	0.46	7.22	±	0.37
HGB	g/dL	14.3	±	0.5	14.7	±	0.3	15.3	±	0.6	14.9	±	0.5
HCT	%	40.1	±	1.8	40.8	±	1.1	43.1	±	1.4**	41.6	±	1.3
PLT	10^3/uL	1071	±	116	1092	±	113	1157	±	190	1084	±	96
MCV	fL	57.7	±	2	57.9	±	2.2	56.8	±	1.9	57.7	±	1.8
MCH	pg	20.6	±	1	20.8	±	0.9	20.1	±	0.7	20.6	±	0.5
MCHC	g/dL	35.8		0.6	36		0.2	35.5		0.3	35.8		0.2
WBC	10^3/uL	7.04	±	2.73	5.88	±	1.38	6.75	±	1.03	5.06	±	0.35

** $p < 0.01$

Table S4. Serum biochemistry values of administration group treated with *Leuconostoc lactis* DMLL10 during a14-day.

Parameter	Units	*Leuconostoc lactis DMLL10*			
		0	2×10^{10}	4×10^{10}	8×10^{10}
No. or rats		10	10	10	10
Male					
ALT	U/L	34.1 ± 2.9	30.2 ± 3.9	28.3 ± 2.6	31 ± 6.2
AST	U/L	96.1 ± 11.9	82.9 ± 12	91.2 ± 14.2	102.3 ± 31.9
ALP	U/L	750.8 ± 146.5	644.2 ± 144.9	697.1 ± 160.9	727.8 ± 369.2
TP	g/dL	5.6 ± 0.1	5.6 ± 0.1	5.4 ± 0.2	5.6 ± 0.2
ALB	g/dL	2.4 ± 0.1	2.4 ± 0.1	2.4 ± 0.1	2.4 ± 0.1
A/G	ratio	0.74 ± 0.04	0.73 ± 0.03	0.77 ± 0.07	0.75 ± 0.02
BUN	mg/dL	12.4 ± 2	11.9 ± 1.2	12.5 ± 1.5	11.8 ± 2.7
Crea	mg/dL	0.36 ± 0.03	0.34 ± 0.02	0.37 ± 0.04	0.33 ± 0.04
T-Chol	mg/dL	81 ± 8	68 ± 6	79 ± 9	72 ± 16
TG	mg/dL	45 ± 26	52 ± 4	39 ± 17	41 ± 15
Glu	mg/dL	112 ± 12	111 ± 20	115 ± 12	103 ± 18
Female					
ALT	U/L	20.1 ± 3.1	20.1 ± 2.3	19.9 ± 4.6	20.9 ± 1.6
AST	U/L	74.7 ± 11.5	82.9 ± 9.9	82.5 ± 14.9	87.2 ± 9.8
ALP	U/L	409.7 ± 37.8	417.4 ± 193.6	414.8 ± 171	418.4 ± 84.3
TP	g/dL	5.8 ± 0.3	5.9 ± 0.3	6 ± 0.3	5.7 ± 0.2
ALB	g/dL	2.7 ± 0.2	2.6 ± 0.2	2.7 ± 0.1	2.6 ± 0.1
A/G	ratio	0.86 ± 0.1	0.82 ± 0.04	0.86 ± 0.07	0.82 ± 0.03
BUN	mg/dL	16.8 ± 6.3	14 ± 1.7	15.4 ± 5.4	14.8 ± 2.6
Crea	mg/dL	0.39 ± 0.06	0.39 ± 0.07	0.37 ± 0.09	0.39 ± 0.06
T-Chol	mg/dL	84 ± 12	75 ± 17	86 ± 8	80 ± 14
TG	mg/dL	18 ± 4	15 ± 6	18 ± 11	16 ± 5
Glu	mg/dL	111 ± 6	106 ± 6	111 ± 11	119 ± 5
